# Clinical efficacy and safety of traditional Chinese medicine Xiao Yao San in insomnia combined with anxiety

**DOI:** 10.1097/MD.0000000000027608

**Published:** 2021-10-29

**Authors:** Jing Hu, Jing Teng, Wanyu Wang, Ni Yang, Haoyu Tian, Wei Zhang, Xiangyu Peng, Jingyu Zhang

**Affiliations:** a First Clinical Medical School, Shandong University of Traditional Chinese Medicine, Shandong, China; b Shandong University of Traditional Chinese Medicine, Shandong, China; c College of Traditional Chinese Medicine, Shandong University of Traditional Chinese Medicine, Jinan, Shandong, China.

**Keywords:** anxiety, insomnia, meta-analysis, randomized controlled trial, systematic review, Xiao Yao San

## Abstract

**Background:**

Patients with long-term insomnia generally experience anxiety and depression. Traditional sleeping pills and anti-anxiety drugs have certain limitations. Xiao Yao San (XYS), a complementary and alternative therapy, has been widely used to treat insomnia combined with anxiety. This study aims to evaluate the efficacy and safety of XYS in the treatment of insomnia combined with anxiety.

**Methods:**

Related literature was retrieved from 8 electronic databases from the establishment time to March 2021. The subjects were diagnosed with insomnia combined with anxiety. We assessed the methodological quality of randomized controlled trials (RCTs) according to the Cochrane Handbook. Data analysis was conducted using RevMan 5.3 software.

**Results:**

The analysis includes 9 RCTs involving 681 patients. Meta-analysis supported that as an auxiliary drug for Western medicine (WM), XYS could enhance the clinical efficacy of insomnia calculated according to the traditional Chinese medicine (TCM) syndrome score scale (risk ratio [RR]: 1.26; 95% confidence interval [CI]: 1.13–1.43; *P* = .0002) and reduced the scores of Hamilton Anxiety Scale (mean difference [MD]: −5.19; 95% CI: −7.78 to −2.60; *P* < .001), Pittsburgh Sleep Quality Index (MD: −3.35; 95% CI: −4.86 to −1.84; *P* < .001), Self-rating Anxiety Scale (MD: −9.38; 95% CI: −10.20 to −8.75; *P* < .001), TCM syndrome score scale for insomnia (MD: −4.45; 95% CI: −6.65 to −2.24; *P* < .001), and TCM syndrome score scale for anxiety (MD: −5.54; 95% CI: −9.48 to −1.6; *P* = .006). The summary analysis also shows that the separate use of XYS was advantageous in reducing the scores of the Hamilton Anxiety Scale (MD: −3.70; 95% CI: −6.31 to −1.09; *P* = .005), Pittsburgh Sleep Quality Index (MD: −1.82; 95% CI: −2.39 to −1.24; *P* < .001), and Self-rating Anxiety Scale (MD: −10.79; 95% CI: −14.09 to −7.49; *P* < .001). The incidence of adverse events with XYS as an ancillary drug or used separately was lower than that in the WM.

**Conclusion:**

Our systematic evaluation and meta-analysis showed that XYS combined with WM or XYS alone was beneficial for improving sleep quality and relieving anxiety. Due to the low methodological quality, small sample size, and significant heterogeneity of RCTs, more rigorous and scientific clinical trials are required to further evaluate the efficacy and safety of XYS.

**PROSPERO registration number:**

CRD42020190613.

## 1 Introduction

Insomnia is a common clinical sleep disorder.^[1]^ Studies have shown that approximately 30% of adults worldwide experience insomnia symptoms.^[2]^ Insomnia has a significant impact on individuals and society, increasing the pressure of individual study, work, and daily life and increasing social medical expenses.^[3]^ In addition, insomnia is considered to be a risk factor for many diseases, including hypertension,^[4]^ cardiovascular disease,^[5]^ obesity, and diabetes.^[6]^ However, patients with long-term insomnia commonly have concomitant diseases,^[7]^ mainly anxiety and depression.^[8]^ Studies have shown that about 40% to 50% of patients with sleep difficulties experience mental disorders,^[9]^ and approximately 75% of patients with anxiety suffer from insomnia or restless sleep.^[10]^ The clinical symptoms of comorbid anxiety and insomnia are much more complicated than those of any single disease.^[11]^ The relationship between insomnia and anxiety is not a simple causal relationship, but a complex bidirectional relationship confirmed through epidemiological studies and systematic reviews.^[12,13]^ Studies have demonstrated that long-term insomnia can induce symptoms of anxiety or aggravate the original anxiety symptoms.^[14]^ The severity of anxiety tends to be positively correlated with the frequency and duration of insomnia.^[15]^ With the alleviation of anxiety symptoms, sleep difficulties can be mitigated or disappear.^[16]^ Therefore, as the number of patients with insomnia and anxiety gradually increases worldwide, more effective treatments need to be determined to reduce the potential life-threatening effects of this disease.

Recent studies have indicated that there are some similar interventions for the treatment of both insomnia and anxiety.^[13]^ In fact, patients’ disease condition can be improved within a short period of time by using traditional sleeping pills and anti-anxiety agents. Unfortunately, the side effects of these drugs cannot be ignored, including nausea, headaches, nightmares, dysphoria, and confusion.^[17]^ Addiction and tolerance resulting from long-term drug use are the main reasons many patients eventually give up treatment.^[17,18]^ In addition, cognitive behavioral therapy for insomnia with anxiety has not yet been fully utilized, as its implementation is restricted by treatment cost, patients’ attitudes, and therapists’ professional level.^[19,20]^ Therefore, in view of the limitations of the above treatment methods, traditional Chinese medicine (TCM) therapy may be an effective alternative and complementary therapy.^[21]^ In TCM, insomnia and anxiety are classified as “insomnia” and “depression syndrome,” according to their clinical manifestations.^[22,23]^ TCM has a long history of treating insomnia combined with anxiety. TCM scholars of successive dynasties have accumulated rich clinical practical experience that is focused on treating many diseases based on syndrome differentiation from the perspective of people's overall concept. This effectively alleviates anxiety symptoms while improving sleep, and achieves the goal of addressing both symptoms and root causes. According to TCM, the pathogenesis of insomnia combined with anxiety is closely related to emotional factors. The common pathogenesis of the diseases is emotional disorder, which then leads to stagnation of liver qi, dysfunction of spleen in transport, and the disorder of qi and blood. According to TCM basic theory, the liver plays an indispensable role in regulating the circulation of qi.^[24]^ By regulating the flow and activity of qi, the liver promotes optimal circulation of blood as well as digestion and metabolism in the spleen and stomach.^[25]^ Liver qi stagnation refers to the pathological changes that the liver lacks the function of regulating the circulation of qi, resulting in the unsmooth circulation of qi and blood. In TCM basic theory, liver qi stagnation is considered to be the basic pathogenesis of insomnia combined with anxiety. Thus, the basic therapeutic principles of TCM involve soothing the liver and relieving depression, nourishing blood, and tonifying the spleen during medication. Traditional Chinese herbal medicine (CHM) is considered the main treatment of diseases in TCM,^[26]^ and has been widely used to treat insomnia combined with anxiety.^[27,28]^ Compared to Western medicine (WM), CHM has the advantages of a more stable curative effect and fewer toxic side effects.^[29]^

The first recorded use of XYS was in Taiping Huimin Heji Ju Fang during the Song Dynasty.^[30]^ The XYS formula contains the following 8 components: Chai Hu (Radix Bupleuri), Dang Gui (Radix Angelicae sinensis), Bai Shao (Radix Paeoniae alba), Bai Zhu (Rhizoma Atractylodis macrocephalae), Fu Ling (Poria), Gan Jiang (Rhizoma Zingiberis recens), Gan Cao (Radix Glycyrrhizae), and Bo He (Herba Menthae).^[31]^ The modified XYS evolved from the original XYS and included additional components such as Mu Dan Pi (Paeonia moutan) and Zhi Zi (Gardenia jasminoides).^[32]^ XYS disperses stagnated liver qi to relieve stagnation, nourish blood, and tonify the spleen.^[33]^ XYS has long been used by TCM doctors to treat anxiety or depression. ^[14,34]^ Many clinical trials and literature reviews reported the beneficial effects of XYS alone or combined with anti-anxiety drugs in the treatment of anxiety symptoms. ^[35–37]^ The anti-anxiety effect of XYS was also supported by a meta-analysis.^[34]^ Recently, evidence from clinical trials showed that XYS was beneficial to insomnia.^[38,39]^ Previous meta-analysis and systematic review showed that XYS alone or as an adjuvant therapy combined with other methods is safe for insomnia patients and can enhance subjective sleep in patients with insomnia.^[40,41]^ In pharmacological studies, kaempferol in Radix Bupleuri can inhibit the activity of monoamine oxidase, prevent the degradation of monoamine neurotransmitters (ie, noradrenaline, 5-hydroxytryptamine, and dopamine) and increase the content of amine substances in the synaptic cleft, thereby exerting the effects of anti-depression and treatment of insomnia.^[42]^ Paeoniflorin in Radix Paeoniae alba and curcumin in Rhizoma Zingiberis recens exerts anti-depression and anti-insomnia effects by regulating the monoamine neurotransmitter system.^[42]^ Animal experiments have shown that the anti-anxiety and neuroprotective effects of XYS may be achieved by attenuating the increase of α-synuclein and corticosterone induced by chronic stress and downregulating protein phosphatase 2A (PP2A) in the hippocampal region.^[43]^ The anti-anxiety effect of XYS and its treatment of insomnia has been strongly verified by both pharmacological research and clinical application. However, to the best of our knowledge, a systematic review and meta-analysis to evaluate the efficacy of XYS in insomnia combined with anxiety has not yet been conducted. Therefore, we evaluated the efficacy and safety of XYS as monotherapy or adjuvant therapy in the treatment of insomnia combined with anxiety to provide a comprehensive basis for clinical differentiation and treatment of insomnia combined with anxiety.

## 2 Methods

The meta-analysis and systematic review of XYS in treating insomnia combined with anxiety were conducted in accordance with the Preferred Reporting Items for Systematic Reviews and Meta-Analyses (PRISMA).^[44]^ In addition, registration of the clinical data was completed on the PROSPERO platform and the corresponding registration number (CRD42020190613) was obtained. This meta-analysis on XYS in the treatment of insomnia combined with anxiety was conducted on the basis of published articles, and no privacy issues were involved. Therefore, the opinions of the ethics committee and patients were not considered.

### 2.1 Search strategy

Multiple electronic databases were utilized to retrieve RCTs on XYS in the treatment of insomnia combined with anxiety, including the China National Knowledge Infrastructure, PubMed, Web of Science, Chinese Scientific Journal Database, EMBASE, Cochrane Library, Chinese Biomedical Literature Database, and Wanfang Database. The databases were searched for related studies published until March 2021. There were no language restrictions on document retrieval. The following search terms were used: (“xiaoyao” OR “xiaoyao san” OR “xiaoyaosan” OR “xiaoyao powder” OR “xiaoyao pill” OR “xiao-yao” OR “xiao yao” OR “xiao yao san” OR “xiao yao pill” OR “xiao yao powder”) AND (“insomnia” OR “chronic insomnia” OR “sleep disturbance” OR “somnipathy” OR “sleep disorder” OR “sleeplessness”) AND (“anxiety” OR “anxiety disorder” OR “anxiety neuroses” OR “neurotic anxiety state”) AND (“randomized controlled trial” OR “controlled clinical trial” OR “randomized” OR “trial”). In order to search for more extensive experimental research, references cited in critical research articles were also manually evaluated.

### 2.2 Study selection

#### 2.2.1 Type of studies

For this study, we included RCTs evaluating the efficacy and safety of XYS or modified XYS for the treatment of insomnia combined with anxiety. RCTs that did not contain sufficient data for analysis were excluded. Studies on the mental conditions of insomnia patients and those on sleep conditions of people with anxiety were also excluded.

#### 2.2.2 Type of participants

RCTs that were conducted in patients with insomnia combined with anxiety were included in the meta-analysis. If the subjects in the individual study suffered from secondary insomnia or only insomnia or only anxiety, then the corresponding RCTs were excluded. Moreover, there were no restrictions on the nationality, age, gender, or race of the subjects. Patients with insomnia combined with anxiety need to have met the diagnostic criteria for any one of the following: Chinese Classification of Mental Disorders Third Edition (CCMD-3),^[45]^ International Statistical Classification of Diseases and Related Health Problems Tenth Revision (ICD-10),^[46]^ Criteria of Diagnosis and Therapeutic Effect of Diseases and Syndromes in Traditional Chinese Medicine (CDTEDSTCM),^[47]^ Guidelines for Diagnosis and Treatment of Common Internal Diseases in Chinese Medicine (GDTCIDCM),^[48]^ and Guidelines for Clinical Research of New Chinese Medicines (GCRNCM).^[49]^

#### 2.2.3 Type of interventions

Various forms (ie, decoction, granules, capsules, and tablets) of XYS or modified XYS were used for specific interventions in each treatment group. One of the following comparisons was included in the RCTs included in the analysis: XYS or modified XYS *vs*. WM; XYS or modified XYS *vs*. placebo; XYS or modified XYS *vs*. lifestyle intervention; XYS or modified XYS and WM versus WM. Any RCTs that used Qigong, Taijiquan, acupuncture, cupping, moxibustion, and massage in combination with XYS or modified XYS were excluded from further analysis. There were no limits on the dose, form, or administration method of XYS or modified XYS. However, in cases wherein XYS or modified XYS was applied together with WM, WM in the experimental and control groups needed to be the same dose. Furthermore, the course of treatment had to last for at least 2 weeks.

#### 2.2.4 Type of outcome measures

We evaluated 4 primary outcomes, including the clinical efficacy of anxiety calculated according to the Hamilton Anxiety Scale (CEAH), the clinical efficacy of insomnia calculated according to the Pittsburgh Sleep Quality Index (CEIP), the clinical efficacy of anxiety calculated according to the TCM syndrome score scale (CEAT), and the clinical efficacy of insomnia calculated according to the TCM syndrome score scale (CEIT). We also assessed several secondary outcomes, such as the scores of Hamilton Anxiety Scale (HAMA), Pittsburgh Sleep Quality Index (PSQI), Self-rating Anxiety Scale (SAS), adverse events (AEs), the TCM syndrome score scale for anxiety (SSSA), the TCM syndrome score scale for insomnia (SSSI), and the treatment emergent symptom scale (TESS).

### 2.3 Data extraction

Studies that were consistent with the research strategies outlined above were independently identified by 2 researchers (JH and JT). The titles and abstracts of the articles were quickly browsed to select RCTs that met the relevant inclusion criteria and were carefully reviewed. Subsequently, relevant information from the study was extracted by 2 researchers (WYW and HYT) for meta-analysis using standard data collection tables (Table 1). Any problems of uncertainty and discrepancy in the data extraction process were resolved through discussion with a third researcher (WZ). The remaining data not reported in the RCTs were obtained by contacting the authors.

**Table 1:** Characteristics of 9 RCTs included in the meta-analysis.

						Intervention				
Studies	Sample size (T/C)	Mean age, y (SD)	Design	Diagnostic criteria for insomnia	Diagnostic criteria for anxiety	Treatment	Control	Duration of intervention, wk	Adverse events	Outcome measures
Deng, 2017^[42]^	50/45	T: 41.66 (3.12)C: 43.0 (6.15)	2 Parallel arms	CCMD-3, GDTCIDCM	CCMD-3, GDTCIDCM	Modified XYS(1 dose/400 mL/day)	Mirtazapine Tablets(30 mg, qd)	8	Y	CEIT, HAMA, PSQI
Li, 2016^[59]^	40/40	T: 42.39 (5.17)C: 42.45 (5.26)	2 parallel arms	CCMD-3, CDTEDSTCM, GCRNCM	CCMD-3, CDTEDSTCM, GCRNCM	Modified XYS(2 mg, tid) + C	Estazolam tablets(1 mg, qd)	6	Y	CEIT, SSSI, SAS
Chen and Zhou2019^[54]^	26/26	T: 44.04 (10.94)C: 46.50 (12.50)	2 Parallel arms	CCMD-3, CDTEDSTCM, GDTCIDCM	CCMD-3, CDTEDSTCM, GDTCIDCM	Modified XYS(4 dose/day) + C	Mirtazapine Tablets(30 mg, qd)	2	Y	CEIT, AEs, PSQI, SSSI
Huang and Xiang, 2014^[52]^	34/28	T: 43.5 (2.5)C: 41.5 (2.5)	2 Parallel arms	CCMD-3	CCMD-3	Modified XYS(1 dose/day)	Estazolam tablets(1 mg, qd)	4	N	CEIT, SAS
Hou, 2014^[55]^	30/30	T: 39 (12.55)C: 42 (11.76)	2 Parallel arms	CCMD-3, CDTEDSTCM, PSQI	CCMD-3, CDTEDSTCM, HAMA	Modified XYS(1 dose/day) + C	Estazolam tablets(1 mg, qd)	4	Y	CEIT, CEAT, CEAH, CEIP, HAMA, PSQI, SSSI, SSSA, TESS
Li, 2019^[56]^	30/30	T: 39.93 (10.27)C: 41.03 (10.31)	2 Parallel arms	CCMD-3, CDTEDSTCM	CCMD-3, CDTEDSTCM	Modified XYS(1 dose/day) + C	Zopiclone tablets(7.5 mg, qd)	4	N	CEAH, CEIP, HAMA, PSQI, SSSI, SSSA
Xue and Guo, 2009^[53]^	44/43	21-68	2 parallel arms	CCMD-3, PSQI	CCMD-3, HAMA	Modified XYS(1 dose/day)	Mirtazapine Tablets(10–45 mg, qd)	5	Y	PSQI, HAMA
Tang, 2018^[57]^	40/40	T: 44.7 (4.1)C: 45.9 (3.2)	2 parallel arms	CCMD-3, PSQI	CCMD-3, HAMA	Modified XYS(1 dose/day) + C	Eszopiclone tablets (2 mg, qd) + ZAC (1.8 g, qd)	15	N	PSQI, SAS
Liu, 2017^[49]^	53/52	T: 26.8 (5.5)C: 43.5 (5.6)	2 parallel arms	CCMD-3, PSQI	CCMD-3, SAS	Modified XYS(1 dose/day) + C	Estazolam tablets(1 mg, qd)	6	N	PSQI, SAS

### 2.4 Assessment of methodological quality

Quality assessment of each RCT was conducted independently by 2 researchers (XYP and JYZ) according to the bias risk assessment tool recommended by Cochrane.^[50]^

The evaluation tools consist of 6 special domains: sequence generation, allocation concealment, blinding (blinding of participants, blinding of investigators, and blinding of outcome assessors), incomplete outcome data, selective outcome reporting, and other sources of bias.^[50]^ An answer to “Yes” indicated a low risk of bias, whereas “No” indicated a high risk of bias. “Unclear” indicated that the risk of bias was unclear or unknown. Different opinions during the process of quality assessment were resolved through discussion with a third researcher (NY).

### 2.5 Data synthesis and analysis

The CEIT, CEAT, CEIP, AEs, and CEAH were considered dichotomous data that allowed calculation of their relative risk (RR) and 95% confidence intervals (CIs). However, the scores of HAMA, PSQI, SSSI, SSSA, TESS, and SAS were considered as continuous data, and therefore, we calculated the mean difference (MD) and 95% CIs for these variables. The heterogeneity between the studies was evaluated using chi-squared statistics. According to the Cochrane System Review Manual, significant heterogeneity was defined as *I*^2^ > 50% or *P* < .05. The random-effects model was then used as a statistical method for data with significant heterogeneity. The fixed-effects model was used for data with insignificant heterogeneity. According to different interventions, data were divided into subgroups for analysis to deal with heterogeneity, and the effect size was combined for each subgroup. The treatment groups were then divided into subgroups with modified XYS, XPWM, and WM treatment, the latter of which served as the control group. The funnel plot method (inverted funnel plot method) was used as a visualization tool to identify publication bias. RevMan software (version 5.3) was used for aggregation and meta-analysis of eligible research data. *P* < .05 was considered statistically significant.

## 3 Results

### 3.1 Search results

**Figure 1:**
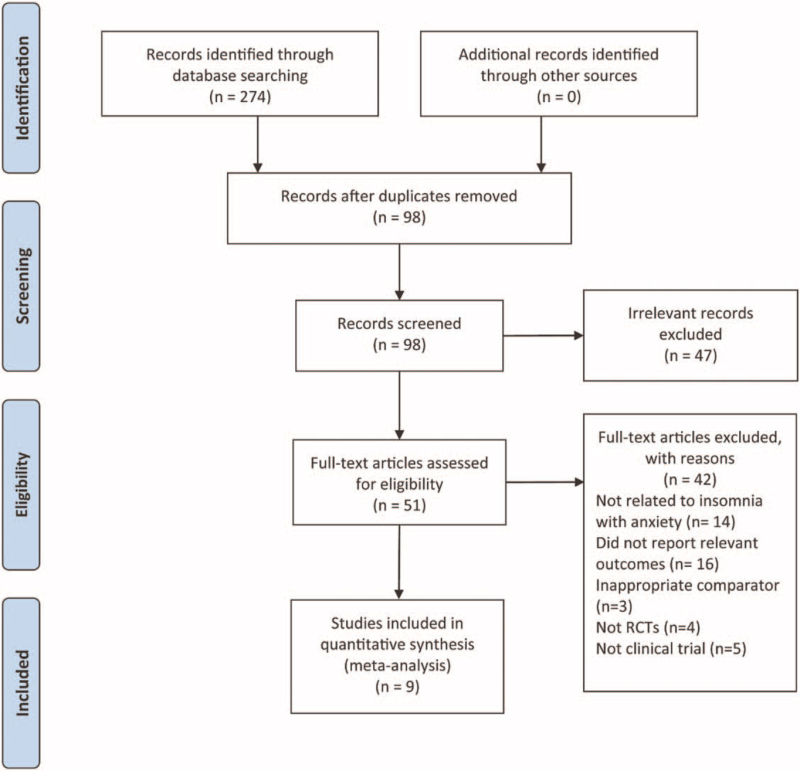
Flow chart of trial selection process.

According to our search strategy, 274 studies were retrieved from 8 electronic databases. Next, we removed 176 studies due to duplication. Among the remaining 98 studies, 47 were excluded by examining the titles and abstracts of each study. After further reading, 42 articles were excluded for several reasons, as shown in Figure 1, for example, not belonging to RCTs on insomnia combined with anxiety, and not reporting related outcome measures. Finally, the meta-analysis involved 9 eligible studies, with the selection process shown in the PRISMA flowchart (Fig. 1).

### 3.2 Study characteristics

The basic characteristics of the 9 RCTs included in the meta-analysis are summarized in Table 1. All clinical trials that met the inclusion criteria were conducted in China and published in Chinese. Additionally, the total sample size included in this meta-analysis was 681, and the sample size of a single study ranged from 52 to 105. Overall, there were 347 patients in the treatment group and 334 patients in the control group. The age of the patients ranged from 18 to 75 years.

Three RCTs compared the treatment group using modified XYS alone and the control group using WM.^[51–53]^ In 6 studies, modified XYS combined with WM was applied to patients in the experimental group, and WM alone was used in the control group.^[54–59]^

### 3.3 Risk of bias

The methodological qualities of the 9 studies are summarized in Table 2. With regard to the generation of random sequences in 9 RCTs, the risk of bias of 5 studies was assessed as low,^[55–59]^ among which the random number table of one study was generated by computer software.^[55]^ The risk of bias of the remaining 4 studies was assessed as “unclear” due to reports that just mentioned “random” but provided insufficient information regarding the methodology of random sequence generation.^[51–54]^ With regard to allocation concealment, 9 studies were assessed as unclear, as it was attributed to the lack of detailed information.^[51–59]^ As for the blind method, one study applied the blind method to both scale operators and outcome evaluators (ie, double-blind method).^[55]^ The remaining studies’ risk of bias were assessed as “unclear” as this section was not described in detail. As none of the studies lost outcome data and all of the predetermined outcomes were reported, and there were no other sources of bias, the corresponding risks of bias were assessed as low.

**Table 2:** Risk of bias in 9 included studies.

Studies	A	B	C	D	E	F	G	H
Deng, 2017^[51]^	?	?	?	?	?	+	+	+
Li, 2016^[59]^	+	?	?	?	?	+	+	+
Chen and Zhou, 2019^[54]^	?	?	?	?	?	+	+	+
Huang and Xiang, 2014^[52]^	?	?	?	?	?	+	+	+
Hou, 2014^[55]^	+	?	?	+	+	+	+	+
Li, 2019^[56]^	+	?	?	?	?	+	+	+
Xue and Guo, 2009^[53]^	?	?	?	?	?	+	+	+
Tang, 2018^[57]^	+	?	?	?	?	+	+	+
Liu et al, 2017^[61]^	+	?	?	?	?	+	+	+

### 3.4 Primary outcome

Among the 9 studies included in the analysis, 6 showed major outcome measures. Hence, a subgroup analysis was conducted for these 6 studies.^[51,52,54–56,59]^

**Figure 2:**
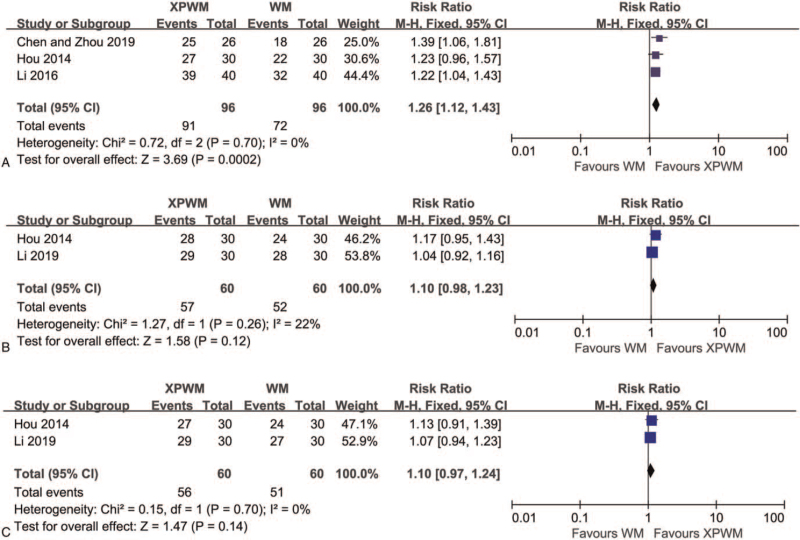
Forest plot comparing XPWM and WM in (A) CEIT, (B) CEAH, and (C) CEIP. CEAH = the clinical efficacy of anxiety calculated according to HAMA, CEIP = the clinical efficacy of insomnia calculated according to PSQI, CEIT = the clinical efficacy of insomnia according to the TCM syndrome scoring scale, CI = confidence interval, HAMA = Hamilton Anxiety scale, WM = Western medicine, XPWM = modified XYS plus WM, XYS = Xiao Yao San.

According to the results of 3 studies on patients with insomnia combined with anxiety,^[54,55,59]^ the improvement effect of XPWM on CEIT was more significant than that of WM (RR: 1.26; 95% CI: 1.13–1.43; *P* = .0002; *I*^2^ = 0%; 3 trials; 192 participants; Fig. 2A).

Two studies reported CEAH and CEIP after treatment with XPWM and WM.^[55,56]^ Meta-analysis indicated that results of CEAH (RR: 1.10; 95% CI: 0.98–1.23; *P* = .12; *I*^2^ = 22%; 2 trials; 120 participants; Fig. 2B) and CEIP (RR: 1.10; 95% CI: 0.97–1.24; *P* = .14; *I*^2^ = 0%; 2 trials; 120 participants; Fig. 2C) showed no significant differences. Only 1 study reported CEAT after treatment with XPWM and WM,^[55]^ and the meta-analysis results also indicated no significant difference (RR: 1.24; 95% CI: 0.94–1.63; *P* = .13; 1 trial; 60 participants).

**Figure 3:**
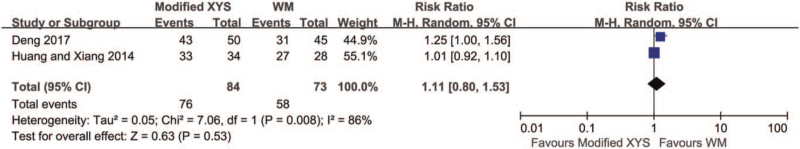
Forest plot comparing Modified XYS and WM in CEIT. CEIT = the clinical efficacy of insomnia according to the traditional Chinese medicine syndrome scoring scale, CI = confidence interval, WM = Western medicine, XYS = Xiao Yao San.

Across the 3 studies comparing the therapeutic results of modified XYS and WM,^[51,53]^ CEIT was reported in 2 studies.^[51,52]^ Pooled analysis indicated that there was no significant difference in the impact of XYS and WM on CEIT (RR: 1.11; 95% CI: 0.80–1.53; *P* = .53; *I*^2^ = 86%; 2 trials; 157 participants; Fig. 3).

### 3.5 Secondary outcomes

**Figure 4:**
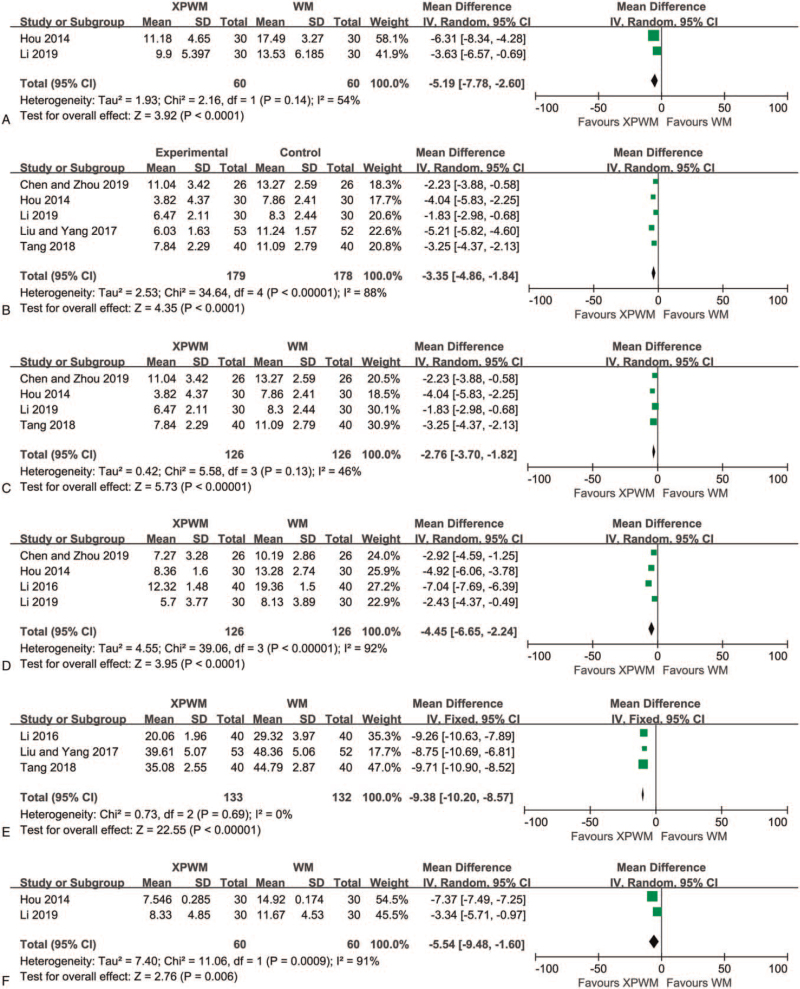
Forest plot comparing XPWM and WM in (A) HAMA, (B) PSQI-1, (C) PSQI-2, (D) SSSI, (E) SAS, and (F) SSSA. CI = confidence interval, HAMA = Hamilton Anxiety scale, PSQI = Pittsburgh Sleep Quality Index, SAS = Self-rating Anxiety Scale, SSSA = the TCM syndrome scoring scale for anxiety, SSSI = the TCM syndrome scoring scale for insomnia, TCM = traditional Chinese medicine, WM = Western medicine, XPWM = modified XYS plus WM, XYS = Xiao Yao San.

HAMA scores before and after treatment were reported in 2 studies.^[55,56]^ The meta-analysis of HAMA scores after treatment was performed because the difference in HAMA scores before treatment was not statistically significant. The results showed that XPWM as a treatment method had a significant effect on reducing HAMA scores (MD: −5.19; 95% CI: −7.78 to −2.60; *P* < .001; *I*^2^ = 54%; 2 trials; 120 participants; Fig. 4A).

Furthermore, PSQI scores after treatment with XPWM and WM were reported in 5 RCTs.^[54–58]^ XPWM was found to be more effective than WM in reducing PSQI scores (MD: −3.35; 95% CI: −4.86 to −1.84; *P* < .001; *I*^2^ = 88%; 5 trials; 357 participants; Fig. 4B). After excluding a low-quality study,^[58]^ XPWM also had a significant reduction effect on PSQI scores compared to WM (MD: −2.76; 95% CI: −3.70 to −1.82; *P* < .001; *I*^2^ = 46%; 4 trials; 252 participants; Fig. 4C).

SSSI scores were also reported in the 4 RCTs.^[54–56,59]^ Meta-analysis results showed that at the end of follow-up, the SSSI scores of the XPWM group were significantly lower than those of the WM group (MD: −4.45; 95% CI: −6.65 to −2.24; *P* < .001; *I*^2^ = 92%; 4 trials; 252 participants; Fig. 4D).

Three RCTs reported SAS scores that were measured after treatment for 4 to 15 weeks.^[57–59]^ Meta-analysis results indicated that, when compared to the WM group, XPWM treatment significantly reduced SAS scores (MD: −9.38; 95% CI: −10.20 to −8.75; *P* < .001; *I*^2^ = 0%; 3 trials; 265 participants; Fig. 4E).

SSSA scores were reported in 2 RCTs.^[55,56]^ Meta-analysis suggested that compared with WM, XPWM had a significant effect on reducing SSSA scores (MD: −5.54; 95% CI: −9.48 to −1.6; *P* = .006; *I*^2^ = 91%; 2 trials; 120 participants; Fig. 4F).

**Figure 5:**
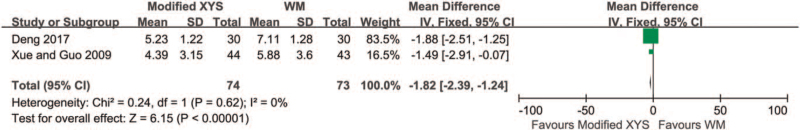
Forest plot comparing modified XYS and WM in PSQI. CI = confidence interval, PSQI = Pittsburgh Sleep Quality Index, WM = Western medicine, XYS = Xiao Yao San.

When comparing modified XYS with WM, 1 study investigated HAMA scores.^[51]^ Meta-analysis results indicated that the separate use of modified XYS was more effective at reducing HAMA scores compared to WM alone (MD: −3.70; 95% CI: −6.31 to −1.09; *P* = .005; 1 trial; 60 participants). Two studies evaluated the influence of modified XYS on the PSQI scores.^[51,53]^ Meta-analysis results indicated that compared to WM, modified XYS had a more significant effect on reducing PSQI scores (MD: −1.82; 95% CI: −2.39 to −1.24; *P* < .001; *I*^2^ = 0%; 2 trials, 147 participants; Fig. 5). One study reported the impact of modified XYS on the SAS scores.^[52]^ Compared to WM, modified XYS led to a significant reduction in SAS scores (MD: −10.79; 95% CI: −14.09 to −7.49; *P* < .001; 1 trial; 62 participants).

AEs were monitored across all 9 RCTs, among which 3 studies reported the incidence and symptoms of AEs before and after the intervention.^[51,54,59]^ The most common adverse events included dry mouth, dizziness, headache, drowsiness, weight gain, and nausea. Meta-analysis results indicated no significant differences in reducing the incidence of adverse reactions between XPWM and WM (RR: 0.17; 95% CI: 0.04–0.72; *P* = .02; *I*^2^ = 0%; 2 trials; 132 participants). There was also no significant difference in reducing the incidence of adverse reactions between the modified XYS and WM (RR: 0.36; 95% CI: 0.07–1.76; *P* = .21; 1 trial; 95 participants). Any reported adverse events were resolved spontaneously without treatment.

TESS scores were reported in 1 article.^[55]^ A small number of slight side effects in the XPWM treatment group were alleviated with time. However, side effects such as dry mouth and constipation were not alleviated more significantly than in the control group. Meta-analysis indicated that XPWM reduced TESS scores more significantly compared to WM alone (MD: −7.40; 95% CI: −8.29 to −6.51; *P* < .001; 1 trial; 60 participants).

### 3.6 Publication bias

**Figure 6:**
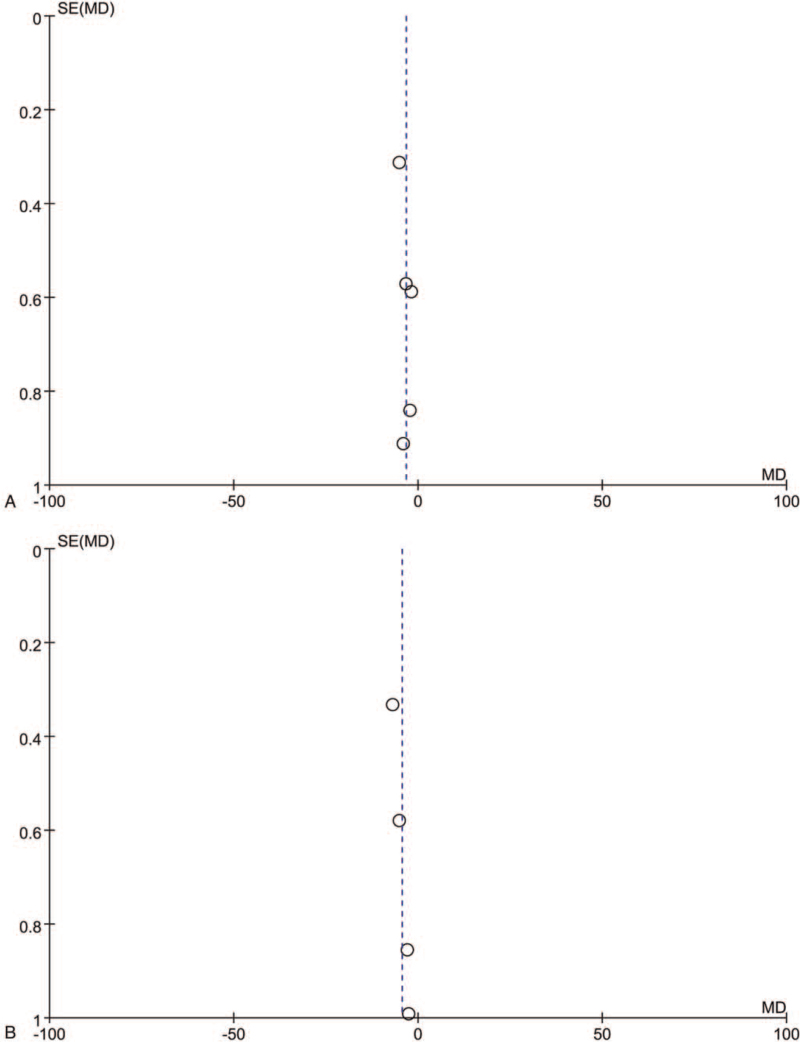
Funnel plots comparing XPWM and WM in (A) PSQI-1 and (B) SSSI. PSQI = Pittsburgh Sleep Quality Index, SSSI = the TCM syndrome scoring scale for insomnia.

Funnel plot analysis was performed on PSQI results of 5 trials and SSSI results of 4 trials to explore publication bias (Fig. 6). The asymmetry of figures indicates publication bias.

## 4 Discussion

Insomnia combined with anxiety is a typical psychosomatic disease, characterized by a series of physical and mental discomforts such as sleep disorder, nervousness, panic and irritability, accompanied by symptoms of autonomic nerve disorders such as dry mouth, flushing and sweating. It gravely affects people 's physical and mental health and quality of life, brings great pain to patients and their families, and causes heavy social and economic burdens. Anxiety is the main cause of insomnia, and insomnia is also the main symptom of anxiety disorder. Modern medicine believes that the causes of insomnia combined with anxiety are various, including biological individual, social factors and psychological factors. At present, the treatment of insomnia combined with anxiety in western medicine is mainly based on drug symptomatic treatment, which has disadvantages such as huge adverse reactions and drug dependence, thus affecting the compliance of patients in medication and treatment. Acupuncture and moxibustion in the treatment of insomnia combined with anxiety has the advantages of rapid onset, exact curative effect, small adverse reactions and easy acceptance by patients, but it is prone to decline in curative effect in the long-term treatment. In contrast, the long-term efficacy of Chinese medicine in the treatment of insomnia with anxiety is better.

The most commonly used Chinese medicinal herbs in modified XYS include Chai Hu (Radix Bupleuri), followed by Dang Gui (Radix Angelicae sinensis), Bai Shao (Radix paeoniae alba), Bai Zhu (Atractylodes ovata), and Fu Ling (Poria Cocos).^[60]^ These herbs are interrelated to form a complex formula of traditional Chinese medicine.^[23]^ The Modified XYS used in most studies included, but was not limited to, these 5 herbs. Chai Hu (Radix Bupleuri) in XYS is a drug that use for dispersing stagnated liver qi to relieve depression and plays the role in regulating liver qi so that it is an indispensable monarch drug in a prescription. Bai Shao (Radix paeoniae alba) and Dang Gui (Radix Angelicae sinensis) are able to nourish the blood and astringe yin, as well as nourish liver and relax tension, serving as a ministerial drug. Bai Zhu (Atractylodes ovata), and Fu Ling (Poria Cocos) can invigorate the spleen and eliminate dampness, enable transportation and transformation, and make qi and blood have the source, which serves as an adjuvant. A little mint is added to disperse the suppressed qi and reach the liver meridian to address the syndrome of heat stagnation, which serves as a conductive drug. The whole compatible prescription is not only for nourishing the liver, but also for aiding liver function, which can benefit both qi and blood, and treat both the liver and spleen. With comprehensive laws and thoughtful medication, XYS is a famous medication for regulating the function of the liver and spleen. In addition, other plants with medicinal value have also been proven to be feasible treatments for insomnia and anxiety.^[29,40]^

Nine RCTs involving 681 participants were reviewed in this meta-analysis and systematic evaluation. The efficacy and safety of using modified XYS as an auxiliary drug therapy or a separate treatment was also evaluated in this study. In general, the results of this meta-analysis validated that XYS is effective in treating insomnia combined with anxiety. As a supplemental drug to WM, XYS improved CEIT scores and reduced the scores of HAMA, PSQI, SSSI, SAS, and SSSA. In addition, summary analysis also indicated that using XYS alone was advantageous in reducing the scores of HAMA, PSQI, and SAS. Furthermore, when modified XYS was combined with WM, the effect of WM was considerably enhanced.

Sensitivity analysis on SSSA showed that the severity of the disease and the intervention in the study were both sources of heterogeneity. In the sensitivity analysis, the positive trend of treatment using modified XYS combined with WM was evident when 2 studies with insufficient methodological qualities were excluded. The separate use of modified XYS also played an active role in reducing the HAMA, PSQI, and SAS scores. Meta-analysis of the subgroups indicated that the combination of modified XYS with WM or the separate use of modified XYS to replace WM are relatively effective drug choices for patients who cannot receive WM treatment or are insensitive to WM treatment. Moreover, the overall effect of XPWM in treating insomnia combined with anxiety was similar to that of the modified XYS.

However, the efficacy and safety of Chinese medicinal herbs combined with WM require further study, as the pharmacokinetics of many medicines can be influenced by ingredients in Chinese medicinal herbs and can cause serious side effects.^[61]^ Furthermore, no serious adverse reactions have been reported due to the use of modified XYS, and the incidence of adverse events remains low. Adverse events are more frequent among people taking WM, but it seems that adding modified XYS to WM can reduce the incidence of side effects. Very few studies have reported on the safety issues of the modified XYS treatment. Therefore, no definite conclusions can be drawn on the efficacy of modified XYS in reducing side effects, and more reliable evidence will be required in the future.

A previous meta-analysis of XYS in the treatment of insomnia showed that XYS is effective and safe for patients with fewer side effects than WM. However, there has been no meta-analysis of XYS in the treatment of insomnia combined with anxiety. Therefore, this meta-analysis is the first to provide evidence on the efficacy and safety of XYS in the treatment of insomnia combined with anxiety. The results of this research will be of significance for the clinical treatment of insomnia combined with anxiety.

This review strictly follows the Preferred Reporting Items for Systematic Reviews and Meta-Analyses (PRISMA). The latest evidence obtained through this meta-analysis can be provided to policymakers and clinicians so that more appropriate drug choices can be made for patients with concurrent insomnia and anxiety. This study also has the following limitations, which should be considered as priorities before applying any conclusions from this study to clinical practice. First, even though our search strategy was comprehensive, articles in non-English languages may have been ignored. Second, the methodological quality of some of the included studies was judged to be defective with regard to random sequence generation, allocation concealment, and blinding. Third, the smaller sample size of the included RCTs may lead to false-positive results, which limits the judgment on the reliability of these results. Fourth, the longest follow-up time for XYS in treating insomnia combined with anxiety was 15 weeks. Hence, the forward clinical efficacy based on follow-up visits was relatively low. Therefore, the safety evidence associated with XYS cannot be generalized to a longer duration of treatment. Fifth, international researchers were unable to access these studies, as none of them were listed in the open database, likely indicating that there is language and publication bias in this meta-analysis. Finally, most studies did not record or report adverse events. Therefore, more clinical data are required to verify the safety of XYS.

## 5 Conclusions

The results from this research have shown that XYS has certain advantages in the treatment of insomnia combined with anxiety and leads to improvement in efficacy and reduction in adverse reactions. XYS should be considered a novel treatment option for insomnia combined with anxiety, and more rigorous and scientific clinical trials are required to further evaluate the long-term efficacy and safety of this medication. In addition, additional studies should focus on the mechanism by which XYS treats insomnia combined with anxiety.

## Acknowledgments

The authors extend their thanks to all the authors of the 9 RCTs included in this meta-analysis.

## Author contributions

**Conceptualization:** Jing Hu, Jing Teng.

**Data curation:** Jing Teng.

**Formal analysis:** Wanyu Wang, Haoyu Tian.

**Funding acquisition:** Wei Zhang, Jingyu Zhang.

**Methodology:** Wanyu Wang, Xiangyu Peng, Jingyu Zhang.

**Project administration:** Haoyu Tian, Xiangyu Peng.

**Software:** Jing Hu, Ni Yang.

**Supervision:** Wei Zhang, Ni Yang, Jing Teng.

**Visualization:** Jing Hu, Jing Teng.

**Writing – original draft:** Jing Hu.

**Writing – review & editing:** Wanyu Wang, Ni Yang, Haoyu Tian.
